# Physical Modeling of Activation Energy in Organic Semiconductor Devices based on Energy and Momentum Conservations

**DOI:** 10.1038/srep24777

**Published:** 2016-04-22

**Authors:** Ling-Feng Mao, H. Ning, Changjun Hu, Zhaolin Lu, Gaofeng Wang

**Affiliations:** 1 School of Computer and Communication Engineering, University of Science and Technology Beijing, Beijing, 100083, China; 2 Microsystems Engineering, Kate Gleason College of Engineering, Rochester Institute of Technology, Rochester, NY 14623, USA; 3 School of Electronics and Information, Hangzhou Dianzi University, Hangzhou, 310018, China

## Abstract

Field effect mobility in an organic device is determined by the activation energy. A new physical model of the activation energy is proposed by virtue of the energy and momentum conservation equations. The dependencies of the activation energy on the gate voltage and the drain voltage, which were observed in the experiments in the previous independent literature, can be well explained using the proposed model. Moreover, the expression in the proposed model, which has clear physical meanings in all parameters, can have the same mathematical form as the well-known Meyer-Neldel relation, which lacks of clear physical meanings in some of its parameters since it is a phenomenological model. Thus it not only describes a physical mechanism but also offers a possibility to design the next generation of high-performance optoelectronics and integrated flexible circuits by optimizing device physical parameter.

In recent years organic semiconductors have gained considerable interest in order to develop low-cost and large-area integrated flexible circuits^1^,^2^,^3^,^4^,^5^,^6^. Understanding of the fundamental physics of organic semiconductor devices such as light-emitting diodes (OLEDs), field-effect transistors (OFETs) and solar cells, accurate modeling of charge transport in these devices is prerequisites to optimize their performances and design organic circuits to realize flexible electronics. Most effort for improving the charge-carrier mobility has been done by optimizing current materials and developing new materials. But high mobility organic semiconductors may not be always valuable, for example, in the fabrication of the organic circuits that are applied to low-end devices^7^ [and the references therein]. Most of existing organic semiconductors are *p*-channel materials and only a small fraction of them are *n*-channel materials^7^ [and the references therein].

The field dependent mobility is thermally activated with the activation energy in an organic semiconductor device. Experiments showed that the carrier mobility depends on the temperature and the gate voltage^8^,^9^,^10^,^11^ [and the references therein]. Thermal activated physical quantities such as the conductance and the mobility in organic semiconductor devices obey an empirical relation, called as the Meyer-Neldel relation^8^,^9^,^10^,^11^ [and the references therein]. The physical meanings of the parameters in the Meyer-Neldel relation are unclear so far because it is a phenomenological model and the charge transport mechanisms are not fully understood yet^8^,^11^. The activation energy is a common factor in models^8^ [and the references therein]. Accurate modeling of thermally activated physical quantities is usually difficult because there exists large variation in the experimental data. One drawback of the Meyer-Neldel relation is that it is a phenomenological model and some of its parameters lose their underlying physics, although its results are consistent with experiments.

The purpose of the present article is to shed some light on the devices physics of electron transport in the organic transistors by virtue of the energy and momentum conservation equations. In particular, a physical model relevant to the activation energy in the organic transistors is proposed. Such a physical model can be applied to describe the experimental relation among thermally activated physical quantities such as the conductance and the mobility in organic semiconductors. The proposed model illustrates the physical origin of a change in the activation energy through its simplicity and analytic nature. In addition, a potential method is developed to calculate the source-drain current and study the relaxation process using the electrical characteristics. The proposed model states that the change in the activation energy in an organic semiconductor device is inevitable by choosing or modulating the energy relaxation time, the momentum relaxation time, the effective electron mass, the doping density (or defect density) and the surface electric field, so that the organic semiconductor device can be reasonably designed to achieve desirable characteristics. This proposed model is not only beautiful because of its physical simplicity but also has important practical applications.

## Results

### Theory

The continuity equation, momentum conservation equation, and energy conservation equation in a semiconductor device can be written as^12^,^13^


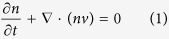



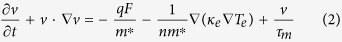



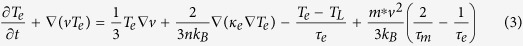


where *n* is the electron density, *τ*_m_ is the momentum relaxation time of electrons, *τ*_e_ is the energy relaxation time of electrons, *v* is the electron velocity, *T*_L_ is the lattice temperature (device temperature), *T*_e_ is the electron temperature, m* is the effective mass of electrons, *k*_B_ is the Boltzmann constant, κe is the thermal conductivity of electron, and *F* is the electric field. For a given point in semiconductor devices, i.e., the space distribution of physical parameters is not considered, only equations (2) and (3) need to be considered and they can be rewritten as


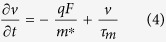



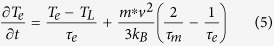


Under equilibrium and a constant electric field, equations (4) and (5) can be rewritten as


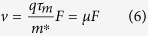



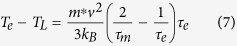


where 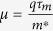 is the electron mobility. Substituting equation (6) into equation (7) leads to


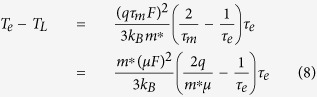


Such an equation implies that, in a field-effect transistor, a lateral electric field in the channel not only results in a drift motion of electrons in the channel, but also changes the random thermal motion (the electron energy or electron temperature). The energy relaxation time can be treated as time and energy independent^14^. For high-energy electrons, the momentum relaxation time is much smaller than the energy relaxation time^15^,^16^,^17^. Under the case of 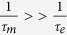, equation (8) can be rewritten as


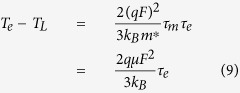


In general, the mobility in MOSFETs is dependent on the electric field^18^,^19^, which can be obtained as


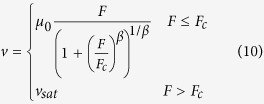


where *μ*_0_ is the electron mobility at low field, *v*_sat_ is the saturation drift velocity, *F*_c_ is the critical field value that is related to the saturation drift velocity by *v*_sat_ = *μ*_0_*F*_c_, and *β* is a fitting parameter. Usually *β* equals to 1 or 2 or the value between 1 and 2. Substituting equation (10) into equations (8) and (9), one can obtain


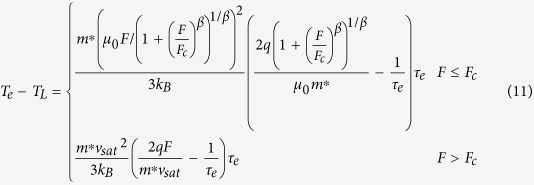



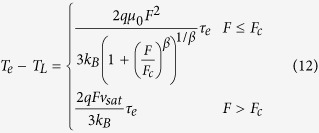


Equations (6, 7, 8, 9, 10, 11, 12) imply that the electrons in semiconductor devices acquire excessive energy from the applied electric field, so that the electron temperature becomes much higher than the lattice temperature.

### Activation energy

In organic electronics, the activation energy is defined as the energy difference between the transport level *E*_μ_ and the Fermi level *E*_F_ in the organic semiconductor^20^, or the energy difference between the trap state and the conduction band edge^21^. The activation energy is one factor to influence hopping transport in organic semiconductors, while other factors are the distance that the carriers have to travel to the adjacent polymer chain and the energy levels of the hopping sites that the carriers have enough energy to hop^22^.

According to equations (6, 7, 8, 9, 10, 11, 12), the electron temperature is much higher than the device temperature in organic semiconductors due to the excessive energy acquired by the electrons from the applied electric field. Equivalently, the effective activation energy by the electrons (i.e., the effective energy difference between the transport level and the Fermi level) is reduced by the excessive electron energy as follows:


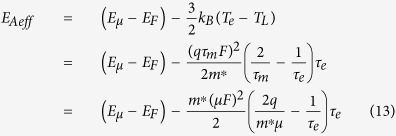


when the momentum relaxation time is much smaller than the energy relaxation time, equation (13) can be simplified as


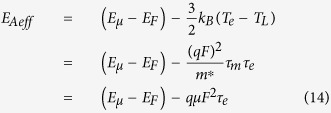


substituting equation (10) into equations (13) and (14), one can obtain


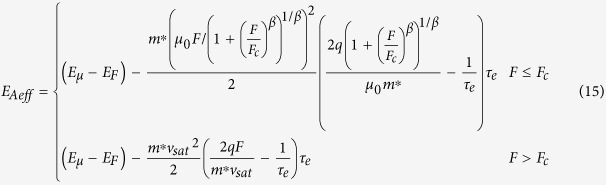



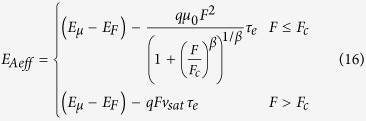


### Source-drain current

For the source-drain current, one can use the equation of the current through the Schottky diode^23^


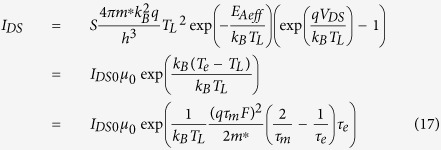


where *h* is Planck constant, *S* is the area, *I*_DS0_ is the current density when the effective activation energy is equal to *E*_μ_ − *E*_F_, and *μ*_0_ is a function of the activation energy but independent of the applied electric field.

Since the standard MOSFET formalism is still applicable to the organic semiconductor field-effect transistors (OFETs)^24^, the source-drain current in OFETs can be written as


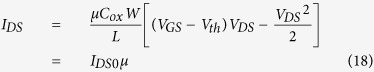


for the linear regime (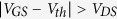),


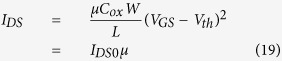


where *C*_ox_ is the capacitance of the gate oxide, *V*_th_ is the threshold voltage, and *W* and *L* are the width and length of the channel in OFETs, respectively.

In equations (17, 18, 19), *I*_*DS*0_ is independent of mobility. Comparing equation (17) with equations (18) and (19), if it is assumed that the increase in the current density is caused by the mobility of carriers due to the activation energy, the mobility can be obtained as


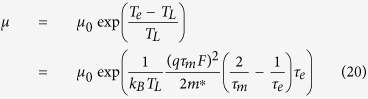


for OFETs, the field dependent mobility using the Meyer-Neldel rule can be obtained as^8^,^9^,^10^,^25^ [and references therein]


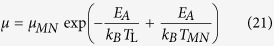


where *T*_MN_ is the Meyer-Neldel temperature, and *μ*_MN_ is the mobility independent of the activation energy and the applied electric field.

If it is assumed that the increase in the current density is caused by the mobility of carriers due to the activation energy, according to^8^,^9^,^10^,^25^ and references therein] and equation (20), and also noting that *μ*_0_ is a function of the activation energy but independent of the applied electric field, one has


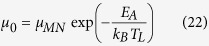


comparing equation (20) with equation (21), one can obtain


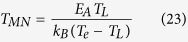



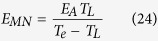


where *μ*_MN_ is the Meyer-Neldel energy. The Meyer-Neldel relation is a phenomenological model and still a topic of discussion^11^. In this proposed model, the Meyer-Neldel energy has a clear physical meaning, i.e., it characterizes the difference of the lattice temperature and the electron temperature using the unit of the activation energy.

### Surface electric field

The above discussions clearly demonstrate that the effective activation energy strongly depends on the applied electric filed. In the following, an analytical model is established to describe the surface electric field along the channel in OFETs.

Noting that the standard MOSFET formalism is still valid to OFETs^24^, the one dimensional (1D) Poisson equation along the channel in OFETs can be written as^23^,^26^,^27^


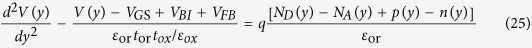


where *V*(*y*) is the electrostatic potential along the channel, *V*_GS_ is the gate-source voltage, *V*_FB_ is the flat band voltage, *V*_BI_ is the built-in potential, *t*_or_ is the thickness of the graphene layer, *t*_ox_ is the thickness of the gate oxide, *q* is the electron charge, *ε*_or_ is the dielectric constant of the organic semiconductor, *ε*_ox_ is the dielectric constant of the gate oxide, *N*_D_(*y*) is the donor density, *N*_A_(*y*) is the acceptor concentrations, *n*(*y*) is the electron density, and *p*(*y*) is the hole density. In an OFET, if the gate dielectric is organic, its relative dielectric constant is low (k ~ 2–4) and hence extremely thin layer are need to obtain low voltage operation (for example operate at 4 V using 10 nm cross-linked polymer and polymer blends), whereas OFET operating below 4 V require high-k dielectric (for example, the anodized TiO_2_, the barium titanate nanocomposite, the relaxor ferroelectric polymer)^28^. The dielectric constant of an organic semiconductor similar to an organic dielectric is also low (k ~ 3–4). For example, for an OFET, the main parameters is the relative dielectric constant of an organic semiconductor as 3.0, the impurity concentration of 1 × 10^16^ cm^−3^, both the gate voltage and drain voltage vary from 0 V to 25 V, the effective electron mass of 114 m_0_, the channel length of 10 μm, and the electron mobility of 0.5 cm^2^/(Vs)^29^. Note that *qV*_*BI*_ is the built-in potential energy difference between the Fermi level of the source and the Fermi level of the channel^23^. For a *p*-type organic semiconductor, one has 
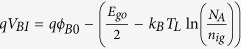
, where *E*_go_ is the band gap of the organic semiconductor, *qϕ*_*B*0_ is the barrier height between the source and the channel, *N*_A_ is the donor doping density in the organic semiconductor, and *n*_io_ is the intrinsic carrier areal concentration of the organic semiconductor. Using the proper boundary conditions to solve equation (25), the electrostatic potential along the channel can be obtained as


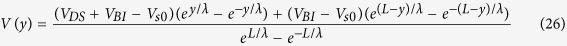


where 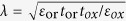, 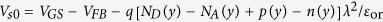, and *V*_DS_ is the source-drain voltage. Therefore, the electric field along the channel is given as


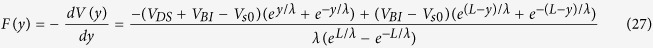


the above equation illustrates that the electric field along the channel is a function of the drain voltage and the gate voltage. The maximum channel electric field can be obtained as


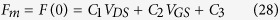


where 
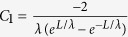
, 
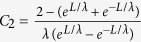
, and *C*_3_ is a parameter independence of the applied voltage. If *L* >> *λ*, one has 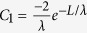 and 
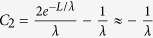
. In the previous studies^27^, *C*_1_, *C*_2_, and *C*_3_ are just fitting parameters. The absolute values of *C*_1_/*C*_2_ from the experimental results have distributed in a wide range such as 0.02/0.00015 ≈ 133, 0.018/0.00022 ≈ 81.8, 0.004/0.00011 ≈ 33.4, 0.001/0.000087 ≈ 11.5, and 0.03/0.009 ≈ 3.3. It implies that, for most cases, the source-drain voltage has a stronger effect on the maximum channel electric field than the gate-source voltage does.

Roland Schmechel *et al*.^30^ deduced the following expression when the absolute value of the source-drain voltage is less than or equal to that of the gate-source voltage:


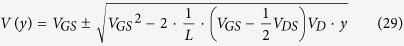


thus, the channel electric field is obtained as


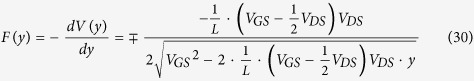


the average channel electric field is given as


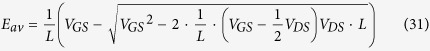


### Relaxation process

If the channel in an OFET is treated as a quantum well, the effective energy relaxation time including all phonon effects can be written as^31^,^32^


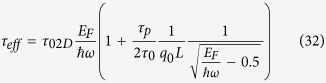


where *τ*_02D_ is the scattering time of two-dimensional (2D) electrons in the channel, *E*_F_ is the Fermi level, *ħω* is the longitudinal optical phonon energy, *τ*_0_ is the electron–phonon scattering time constant, *q*_0_ is the phase matching wave vector, *L* is the channel width, and *τ*_p_ is the phonon lifetime.

Using perturbation theory, the momentum relaxation time caused by elastic acoustic deformation potential scatter can be written as^33^


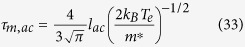


where 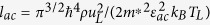 is the carrier mean free path, *ρ* is the mass density, *u*_*L*_ is the longitudinal sound velocity, and *ε*_*ac*_ is the deformation potential constant.

## Discussion

According to equation (16), the effective activation energy is 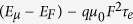 for *F*_C_ >> *F*, while the effective activation energy is 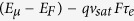 for *F*_C_ < *F*. Note that the maximum electric field is linearly dependent on the source-drain voltage and the source-gate voltage according to equation (28). This implies that the effective activation energy is linearly dependent on the square of the source-gate (drain) voltage in the regime of the low electric field, whereas it is linearly dependent on the source-gate (drain) voltage in the saturation region of OFET.

**Figure 1:**
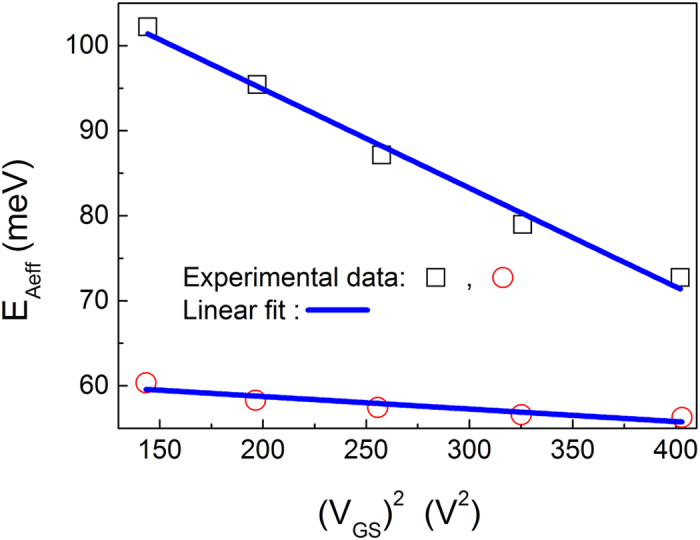
Activation energy as a function of the square of the gate voltage. The open circles and squares correspond to the experimental values of the activation energies for single-grain and polycrystalline OFETs, respectively. Experimental data is from^34^.

Figure 1 clearly shows that the activation energies for both a single-grain pentacene field-effect transistor and a polycrystalline pentacene field-effect transistor are linearly dependent on the square of the gate voltage (Experimental data is from^34^). Such a linear relation between the activation energy and the square of the gate voltage is predicted by equations (16) and (28). Figure 1 illustrates that the proposed model is valid to describe electron transport in pentacene field-effect transistors.

**Figure 2:**
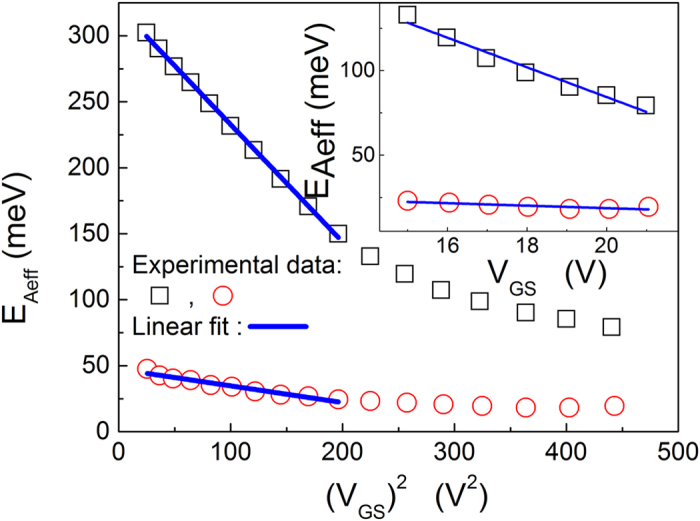
Activation energy as a function of the gate voltage and its square. The open circles and squares correspond to the experimental values of the activation energies in C_60_-based organic FETs for 170 K–77 K and 300 K–190 K, respectively. Experimental data is from^8^.

Figure 2 and its inset depict how the effective activation energy changes with the gate voltage in vacuum evaporated C_60_-based organic FETs. This figure clearly shows that the effective activation energy is linearly dependent on the square of the gate voltage in the regime of low electric field, whereas it is linearly dependent on the gate voltage in the regime of high electric field since the drift velocity reaches saturation. It can be clearly concluded from Fig. 2 that *E*_*eff*_ ∝ −*F*^2^ when V_GS_ < 14 V and *E*_*eff*_ ∝ −*F* when V_GS_ > 14 V. The proposed model predicts that the effective activation energy is linearly dependent on the square of the source-gate (drain) voltage in the regime of the low electric field, whereas it is linearly dependent on the source-gate (drain) voltage in the saturation region. That is, the proposed model agrees well with the experimental data.

**Figure 3:**
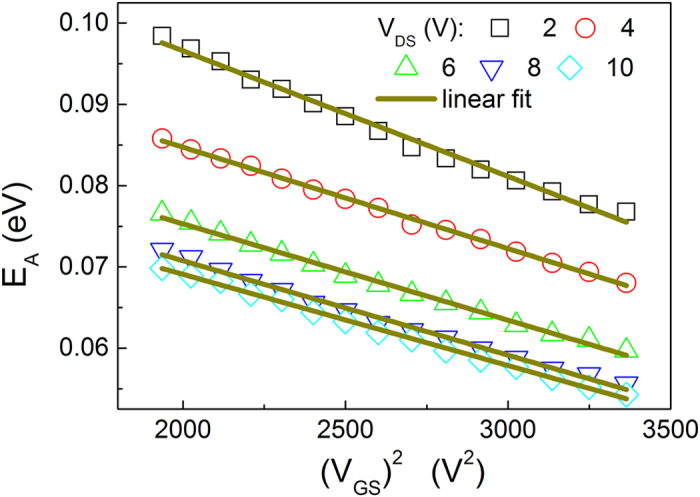
Activation energy as a function of the square of the gate voltage under different source-drain voltages. Experimental data is from^35^.

Figure 3 further shows that the activation energy is linearly dependent on the square of the gate voltage in fullerene C_60_ OFETs under different source-drain voltages. According to the above discussion, equations (13, 14, 15, 16) and equation (28), the activation energy is always linearly dependent on the square of the gate voltage if the mobility keeps constant. It can be concluded from Fig. 3 that the activation energy is linearly dependent on the square of the gate voltage.

**Figure 4:**
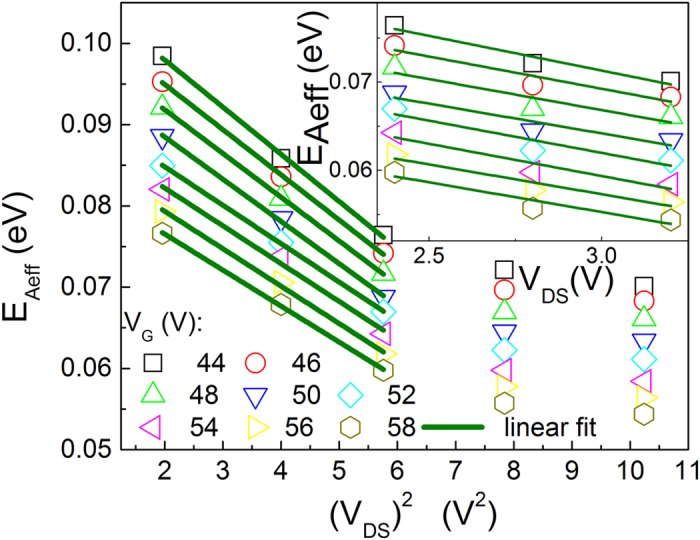
Activation energy as a function of the source-drain voltage and its square under different gate voltages. Experimental data is from^35^.

Figure 4 and its inset illustrate how the source-drain voltage affects the activation energy in fullerene OFETs. According to equation (28), the maximum electric field is linearly dependent on the source-drain voltage. According to equations (13, 14, 15, 16) and equation (28), it can also be concluded that the effective activation energy is linearly dependent on the square of the source-drain voltage in the regime of low electric field (the case of the channel field less than the critical field value that implies the electron velocity depends on the electric field), whereas it is linearly dependent on the source-drain voltage in the regime of high electric field since the drift velocity reaches saturation (the case of the channel field larger than the critical field value that implies the electron velocity reach saturation). There are two type relations, one is a quadratic dependent relation at low electric field, and the other is a linear relation at high electric field (both relations have been given in Equation16). For the linear relation, according to Eq. 7, the electron can get an energy from an applied electric field, which is 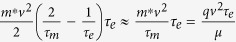. Considering the definition of mobility, 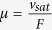 when electron reach saturation, and the energy is 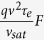, and the slope of a linear model is determined by the saturation velocity and the energy relaxation time. Both relations between the activation energy and the source-drain voltage can be clearly observed in Fig. 4. Now, it is time to shed some light on why there always is a linear dependent relation between the activation energy and the square of the gate voltage under different source-drain voltages. Since the parameters, such as oxide thickness, cannot be found in ref. 35, equation (28) cannot be adopted to calculate the channel electric field. However, the channel length of 35 μm and its width of 2 mm have been given, implying that the channel electric field can be calculated by using equation (31).

**Figure 5:**
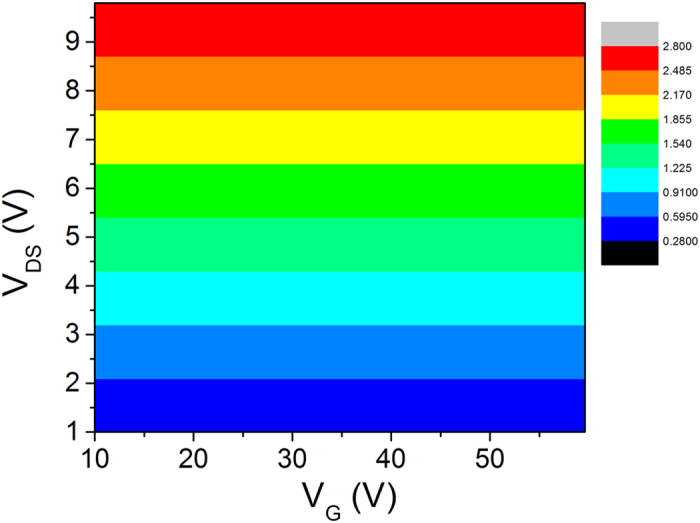
Contour of the channel electric field (unit: kv/cm) affected by the gate voltage and the source-drain voltage.

Figure 5 shows how the gate voltage and the sour-drain voltage affect the channel electric field. This figure clearly illustrates that the channel electric field at a given gate voltage rapidly increases with the source-drain voltage for the case as in ref. 35, whereas it increases little with the gate voltage. Because the gate voltage has little impact on the channel electric field, the mobility can be seen as a constant. It is the reason why the activation energy is always linearly dependent on the square of the gate voltage.

**Figure 6:**
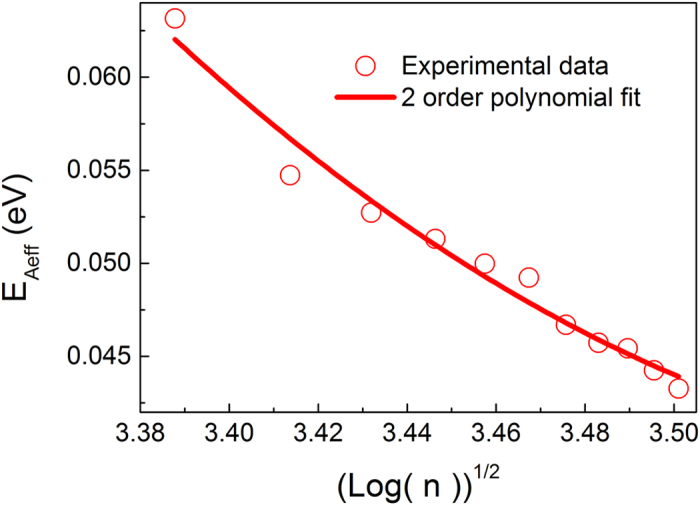
Activation energy as a function of the square root of the nature logarithm of the carrier concentration. The open circles correspond to the experimental values of the activation energy in diketopyrrolopyrrole-based transistors. Experimental data is from^36^.

Figure 6 depicts how the carrier concentration affects the activation energy. Experimental results show that the activation energy decreases with increasing carrier concentration in PDPPFC24-TVT thin film transistors^36^. Its carrier concentration *n* is given as 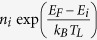 if such a donor-acceptor material behaves as *n*-type organic semiconductor^23^, where *E*_i_ and *n*_i_ are the intrinsic Fermi level and the intrinsic carrier concentration, respectively. Thus, 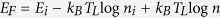. According to equation (32), 
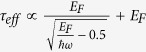
. If *E*_F_ >> 2*ħω*, 
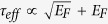
. Note that 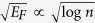 if the reference zero energy is set as 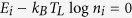. This means that 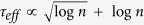. According to equation (13), the reduction in the activation energy is linearly dependent on the energy relaxation time. This implies that the effective activation energy decreases with increasing carrier concentration and obeys 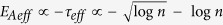. Figure 6 clearly shows that such a 2-order polynomial fitting agrees well with the experimental data. Similarly, one can obtain 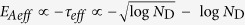. The experimental studies on the impact of the doping concentration on the electrical properties for an *n*-type doped material showed that the activation energy decreases with the doping concentration^20^. The experimental results further validate the proposed physical model.

The effective carrier mass of the value between 0.9 m_0_ and 1.0 m_0_ have been obtained in the polymer film^37^, where m_0_ is free electron mass. The effective electron mass m* of monolayer C_60_ extracted from experiments was obtained as 3 m_0_, whereas the simulation data corresponded to 1 m_0_^38^. The effective electron masses of 0.63 m_0_, 0.59 m_0_, and 1.3 m_0_ were obtained for one-dimensional C_60_ chain, [2 + 2] C_60_ polymer and face center cubic C_60_ crystal^39^. The effective electron mass in pentacene can be obtained as 10^4^ m_0_^40^. Note that m* characterizes the extent of localization or delocalization of the electron wave function in the surface plane. In other words, the states with small effective electron mass are delocalized, whereas those with large effective mass are spatially localized. The momentum relaxation time can be obtained from several fs to tens fs in polymer-fullerene blends^41^, and 46 fs in ultra-thin film perylene tetracarboxylic dianhydride^42^. The energy relaxation times of 36 fs and 491 fs in conjugated polymers have been observed^43^.

**Figure 7:**
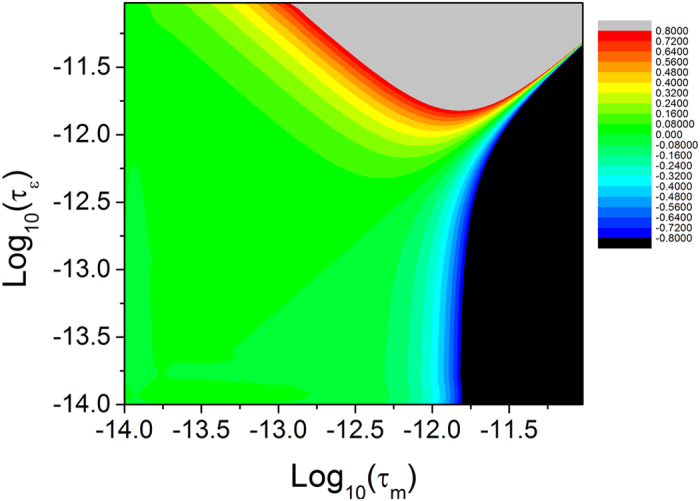
Contour of the change in the activation energy (unit: eV) affected by the momentum relaxation time and the energy relaxation time. The channel electric field of 20 kv/cm and the free electron mass are used in the calculation.

Figure 7 shows how the momentum relaxation time and the energy relaxation time (in the unit of second) affect the effective activation energy. This figure clearly shows that the effective activation energy keeps a constant when both the momentum relaxation time and the energy relaxation time are sufficiently small (e.g., less 1 fs). When the energy relaxation time is larger than the momentum relaxation time, the effective activation energy becomes smaller, and vice versa. Note that negative values in the contour map in Fig. 7 represent the increase of the activation energy.

**Figure 8:**
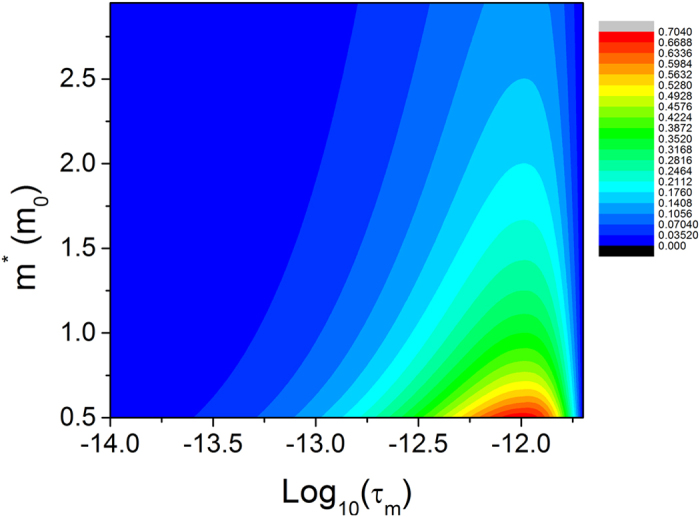
Contour of the change in the activation energy (unit: eV) affected by the momentum relaxation time and the effective electron mass. The channel electric field of 20 kv/cm and the energy relaxation time of 1 ps are used in the calculation.

Figure 8 depicts the contour of the change in the activation energy caused by the effective mass and the momentum relaxation time. It is evident from Fig. 8 that a smaller effective electron mass results in a larger change in the activation energy.

**Figure 9:**
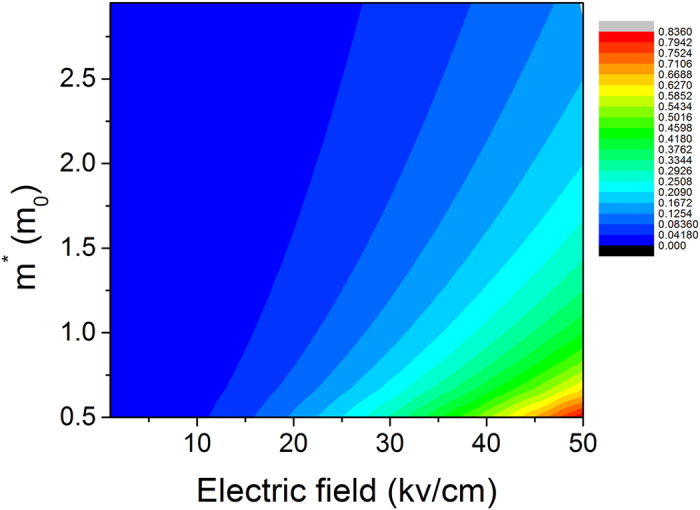
Contour of the change in the activation energy (unit: eV) affected by the channel electric field and the effective electron mass. The momentum relaxation time of 100 fs and the energy relaxation time of 1 ps are used in the calculation.

Figure 9 illustrates how the electric field and the effective electron mass affect the change in the activation energy. It can be clearly observed from Fig. 9 that a larger electric field results in a larger change in the activation energy.

**Figure 10:**
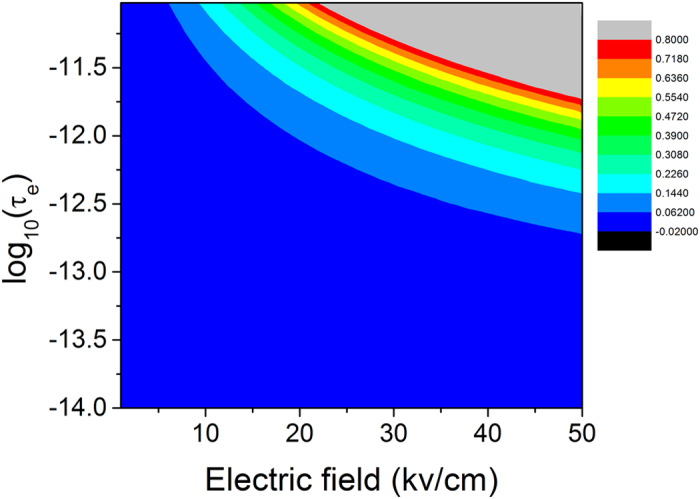
Contour of the change in the activation energy (unit: eV) affected by the channel electric field and the energy relaxation time. The momentum relaxation time of 100 fs and the free electron mass are used in the calculation.

Figure 10 plots the contour of the change in the activation energy caused by the electric field and the energy relaxation time. It can be clearly observed from Fig. 10 that both the energy relaxation time and the electric field can have a large effect on the activation energy. All these observations imply that the energy relaxation and the momentum relaxation of channel electrons should be taken into account for modeling electric characteristics in organic semiconductor devices. It is also found that the activation energy may be relatively independent of the gate voltage if the energy relaxation time and the momentum relaxation time are very small (e.g., both less than 1 fs). These results are of high importance for the design of new OFET materials.

## Conclusion

In conclusion, the effects of the energy relaxation and the momentum relaxation of electrons in organic semiconductors on the effective activation energy have been theoretically investigated and physical modeled. The theoretical calculations agree well with experimental data reported in refs 8,34, 35, 36. The energy relaxation of electrons in organic semiconductors can result in a high electron temperature, thereby causing reduction in the effective activation energy. Based on the energy and momentum conservation equations, a physical model has been built to describe the gate voltage dependent activation energy in organic semiconductor devices observed in the experiments. Effects of the parameters such as the momentum relaxation time, the energy relaxation time, the electric field, and the effective electron mass on the effective activation energy have been analyzed in detail. It revealed that all these parameters have impacts on the effective activation energy. According to the proposed model, the effective activation energy can be linear functions of either both the gate voltage and the drain voltage themselves or their squares, depending on whether the electron velocity is below saturation or not. By assuming 
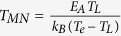
 (i.e., implying 
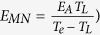
, the activation energy in the proposed model leads to the same mathematical form as described in the Meyer-Neldel model. Note that the Meyer-Neldel model is a phenomenological model. By contrast, the proposed model is a physical model, in which the physical meanings of all parameters are clear, thereby leading to a superior model over the Meyer-Neldel model. By the proposed physical model, the device performance can be readily optimized via changing the device parameters. Through this study, it is evident that the effects of the energy relaxation and the momentum relaxation of electrons in organic semiconductor devices should be seriously included in their electrical characterizations.

## Additional Information

**How to cite this article**: Mao, L.-F. *et al*. Physical Modeling of Activation Energy in Organic Semiconductor Devices based on Energy and Momentum Conservations. *Sci. Rep.* **6**, 24777; doi: 10.1038/srep24777 (2016).
